# Aberrant NRF2-Dependent Cytoprotection Subverts Stress Homeostasis and Promotes Tumor Progression

**DOI:** 10.3390/cimb48101022

**Published:** 2026-10-03

**Authors:** Gaoqing Feng, Yuhong Yang, Huahua Wang, Mao Du, Qin Xia, Lei Dong

**Affiliations:** Advanced Technology Research Institute, State Key Laboratory of Hearing and Balance Science and Key Laboratory of Molecular Medicine and Biological Diagnosis and Treatment ( Ministry of Industry and Information Technology), School of Life Science, Beijing Institute of Technology, Beijing 100081, China

**Keywords:** NRF2, KEAP1-CUL3, GSK3/β-TrCP, SMURF2, stress adaptation, cancer survival

## Abstract

Nuclear factor erythroid 2-related factor 2 (NRF2) coordinates antioxidant defense, metabolism, iron homeostasis, and proteostasis to support cellular adaptation to stress. Kelch-like ECH-associated protein 1 (KEAP1)-Cullin 3 (CUL3) continuously targets NRF2 for proteasomal degradation. Oxidative or electrophilic stress modifies sensor cysteines in KEAP1, transiently attenuating degradation and allowing NRF2 to stabilize and accumulate in the nucleus. Complementary KEAP1-independent mechanisms limit excessive or prolonged NRF2 activity, including glycogen synthase kinase 3 (GSK3)/β-transducin repeat-containing protein (β-TrCP)-mediated degradation and SMAD-specific E3 ubiquitin-protein ligase 2 (SMURF2)-mediated nuclear clearance. In cancer, genetic, epigenetic, and signaling alterations can disable these restraints and sustain NRF2 activity. Persistent NRF2 signaling increases cancer-cell stress tolerance by supporting antioxidant defense, metabolic adaptation, proteostasis, and resistance to therapy. This review examines the spatial and context-dependent control of NRF2 by KEAP1-CUL3, GSK3/β-TrCP, and SMURF2 and considers how failure to terminate cytoprotective signaling supports tumor progression.

## 1. Introduction

Nuclear factor erythroid 2-related factor 2 (NRF2) coordinates cytoprotective programs that enable cells to adapt to stress [1,2]. Its effects depend on the magnitude and duration of activation and on cellular context. Under basal conditions, NRF2 abundance remains low, limiting inappropriate cytoprotective signaling. Oxidative or electrophilic stress rapidly stabilizes NRF2, enabling a transient response that limits damage and supports adaptation. In normal cells, controlled NRF2 activation protects against stress-induced injury and contributes to chemoprevention. Cancer cells, however, can co-opt persistent NRF2 activity to withstand the oxidative and metabolic demands of the tumor microenvironment [3]. Temporal control of NRF2 activation is therefore essential to ensure effective stress protection without disrupting normal cellular homeostasis.

Ubiquitin-dependent degradation is a major mechanism controlling NRF2 abundance and activity [4]. Several E3 ubiquitin ligases target NRF2 for degradation, including the Kelch-like ECH-associated protein 1 (KEAP1)-Cullin 3 (CUL3) complex, the SCF^β-TrCP^ complex, HMG-CoA reductase degradation protein 1 (HRD1, also called synoviolin 1 [SYVN1]), CRL4^DCAF11^ (also called CRL4^WDR23^), and SMAD-specific E3 ubiquitin-protein ligase 2 (SMURF2) [4,5,6,7]. HRD1/SYVN1 promotes NRF2 degradation through the Neh4 and Neh5 domains during endoplasmic reticulum (ER) stress, whereas CRL4^DCAF11^ recognizes the DIDLID motif in Neh2. Both provide KEAP1-independent routes of degradation [8,9]. Their physiological contributions remain less fully defined and appear to vary with tissue, stress state, and subcellular compartment [4].

**Figure 1:**
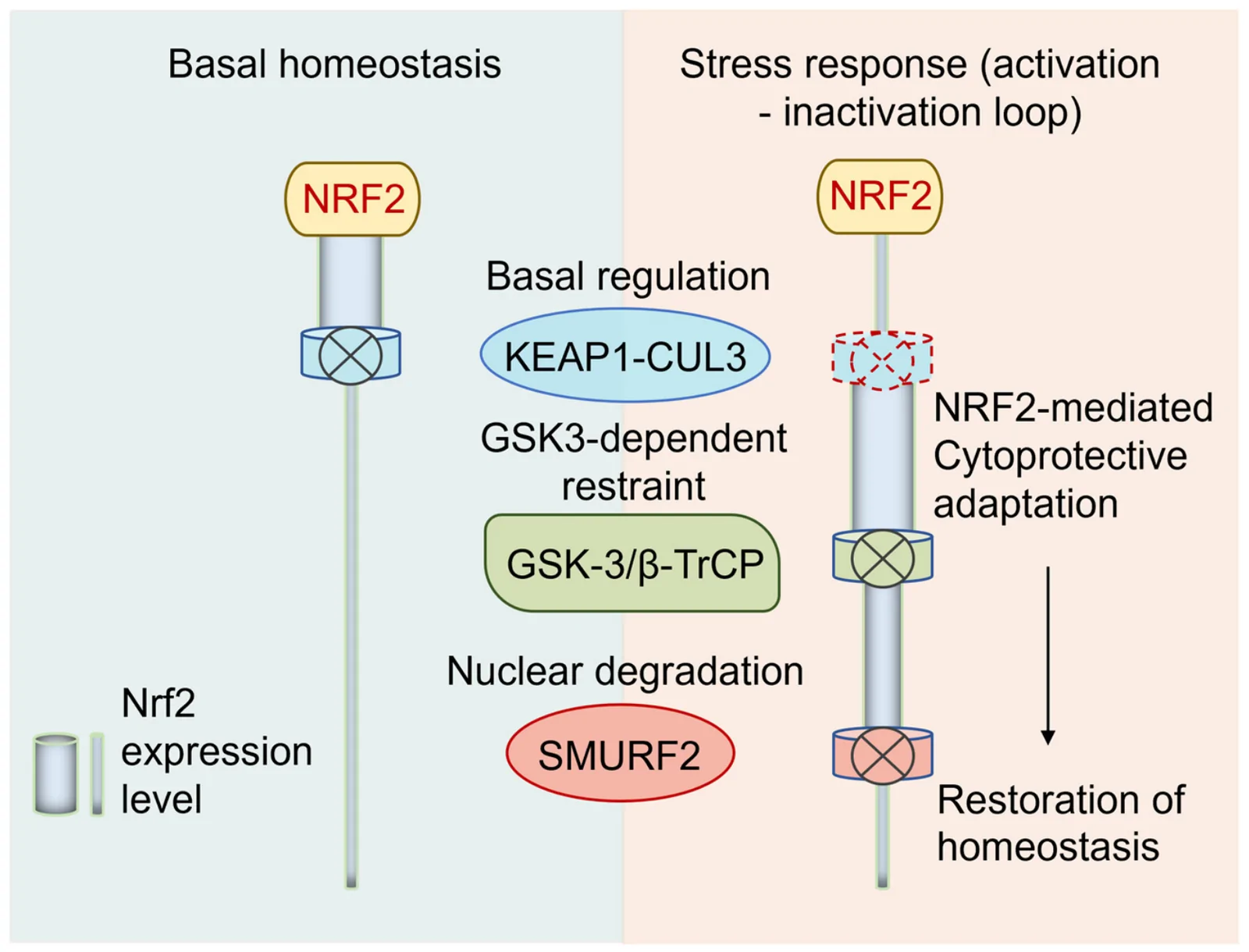
Complementary mechanisms of NRF2 degradation maintain cellular homeostasis. Under basal conditions, the KEAP1-CUL3-RBX1 E3 ubiquitin ligase complex constitutively targets NRF2 for ubiquitination and proteasomal degradation, maintaining low NRF2 abundance. Oxidative or electrophilic stress attenuates this process, permitting NRF2 stabilization and accumulation. GSK3/β-TrCP provides additional KEAP1-independent restraint, whereas SMURF2 directly promotes nuclear NRF2 degradation. These mechanisms limit NRF2 signal duration and support the restoration of homeostasis. These mechanisms represent complementary regulatory routes; their temporal order during a single stress response has not been established.

Transcriptional, epigenetic, and other post-translational mechanisms also regulate NRF2. This review focuses on E3 ligase-mediated degradation because it directly controls NRF2 abundance and signal persistence (Figure 1). Earlier models emphasized the KEAP1-CUL3 and glycogen synthase kinase 3 (GSK3)/β-transducin repeat-containing protein (β-TrCP) pathways [10] but did not fully explain the direct removal of NRF2 that had accumulated in the nucleus. The recent report of SMURF2-mediated NRF2 nuclear degradation provides a potential mechanism for this process.

Recent reviews, including Chen et al. (2026), have broadly examined NRF2 regulation, downstream transcriptional programs, disease roles, and pharmacological modulation [10]. Here, we take a degradation-centered perspective, examining the complementary roles of KEAP1-CUL3, GSK3/β-TrCP, and SMURF2 in controlling NRF2 abundance, persistence, and nuclear activity. This perspective helps explain how cytoprotection remains transient during physiological stress and how failure of signal termination can support cancer-cell survival.

Relevant literature was identified through searches of PubMed and the reference lists of pertinent articles, with the search last updated on 21 September 2026. Studies were selected for their relevance to NRF2 regulation, degradation mechanisms, and cancer, with particular attention to primary mechanistic studies and recent reviews.

## 2. NRF2 in Cellular Homeostasis

NRF2 maintains cellular homeostasis through a broad transcriptional program that coordinates multiple cytoprotective processes [11,12]. In antioxidant defense, NRF2 heterodimerizes with small MAF proteins (sMAFs) and binds antioxidant response elements (AREs) in target-gene regulatory regions [13]. Established targets include NAD(P)H quinone dehydrogenase 1 (NQO1), glutamate–cysteine ligase catalytic subunit (GCLC), and heme oxygenase 1 (HMOX1/HO-1), which increase the capacity to neutralize reactive species and electrophiles [14]. NRF2 also supports metabolic adaptation by inducing pentose phosphate pathway enzymes, including glucose-6-phosphate dehydrogenase (G6PD), phosphogluconate dehydrogenase (PGD), transketolase (TKT), and transaldolase 1 (TALDO1), as well as the NADPH-producing enzyme malic enzyme 1 (ME1), thereby redirecting glucose metabolism and increasing NADPH availability [15]. In addition, NRF2 contributes to iron homeostasis by regulating ferritin metabolism, ferroportin, and heme turnover, thereby modulating the labile iron pool and iron-driven oxidative damage [10,16]. In parallel, NRF2 supports proteostasis through both the ubiquitin–proteasome and autophagy–lysosome systems. It increases the expression of proteasome components and autophagy-related genes, including *SQSTM1* and *ULK1*, thereby facilitating the clearance of damaged and misfolded proteins [17,18,19]. Because these programs strongly influence cell survival, multiple ubiquitin-dependent mechanisms constrain NRF2 abundance and signal duration.

### 2.1. KEAP1-CUL3 Couples Constitutive NRF2 Degradation to Stress-Responsive Stabilization

Under basal conditions, NRF2 is constitutively degraded by the KEAP1-CUL3-RING-box protein 1 (RBX1) E3 ubiquitin ligase complex, the best-characterized regulator of NRF2 stability. KEAP1 serves as the substrate adaptor, recruiting newly synthesized NRF2 for lysine 48 (K48)-linked polyubiquitination and proteasomal degradation. Reported NRF2 half-lives are approximately 10–30 min, depending on cellular and experimental context [14,20]. The BTB (Broad complex, Tramtrack, and Bric-à-brac) domain of KEAP1 recruits CUL3, whereas its Kelch domain recognizes the ETGE and DLG motifs in the NRF2 Neh2 domain. Simultaneous engagement of the high-affinity ETGE motif and lower-affinity DLG motif positions NRF2 for efficient ubiquitination by the KEAP1-CUL3-RBX1 complex [21]. This rapid turnover maintains low basal NRF2 abundance.

Oxidative or electrophilic stress modify specific KEAP1 cysteines and reduce its ability to promote NRF2 ubiquitination [22]. KEAP1 does not rely on a single sensor; Cys151, Cys273, and Cys288 act as distinct or cooperative sensors for different electrophiles. Cys151 is a principal sensor for several NRF2 activators, including sulforaphane [23], whereas Cys273 and Cys288 are required for basal KEAP1 repression and also participate in electrophile sensing [24]. Cys288 is a preferential sensor for 15-deoxy-Δ12,14-prostaglandin J2 (15d-PGJ2), whereas electrophiles such as 4-hydroxy-2-nonenal (4-HNE) engage multiple cysteines [23]. Additional combinations, including Cys226, Cys613, Cys622, and Cys624, contribute to hydrogen peroxide sensing [25]. Although individual stressors engage different sensor residues, these modifications converge on attenuation of KEAP1-dependent NRF2 ubiquitination.

Stress-induced KEAP1 modification does not necessarily cause complete dissociation of NRF2. In the classical hinge-and-latch model, modification weakens the lower-affinity DLG interaction while preserving the high-affinity ETGE interaction, leaving NRF2 bound to KEAP1 but preventing productive ubiquitination [26]. More recent NMR analyses showed that representative electrophilic NRF2 inducers do not disrupt either the DLGex–KEAP1 or ETGE-KEAP1 interaction, indicating that electrophile-induced NRF2 activation does not necessarily require classical DLGex latch dissociation [27]. Newly synthesized NRF2 then escapes degradation, accumulates, and translocates to the nucleus, where it heterodimerizes with sMAFs and activates ARE-dependent transcription.

A noncanonical, cysteine-independent mechanism also attenuates KEAP1-dependent NRF2 degradation. p62 binds the Kelch domain of KEAP1 through its KEAP1-interacting region and competes with NRF2, thereby reducing NRF2 ubiquitination. This mechanism becomes particularly prominent when autophagic flux is impaired, and p62 accumulates [28,29].

**Figure 2:**
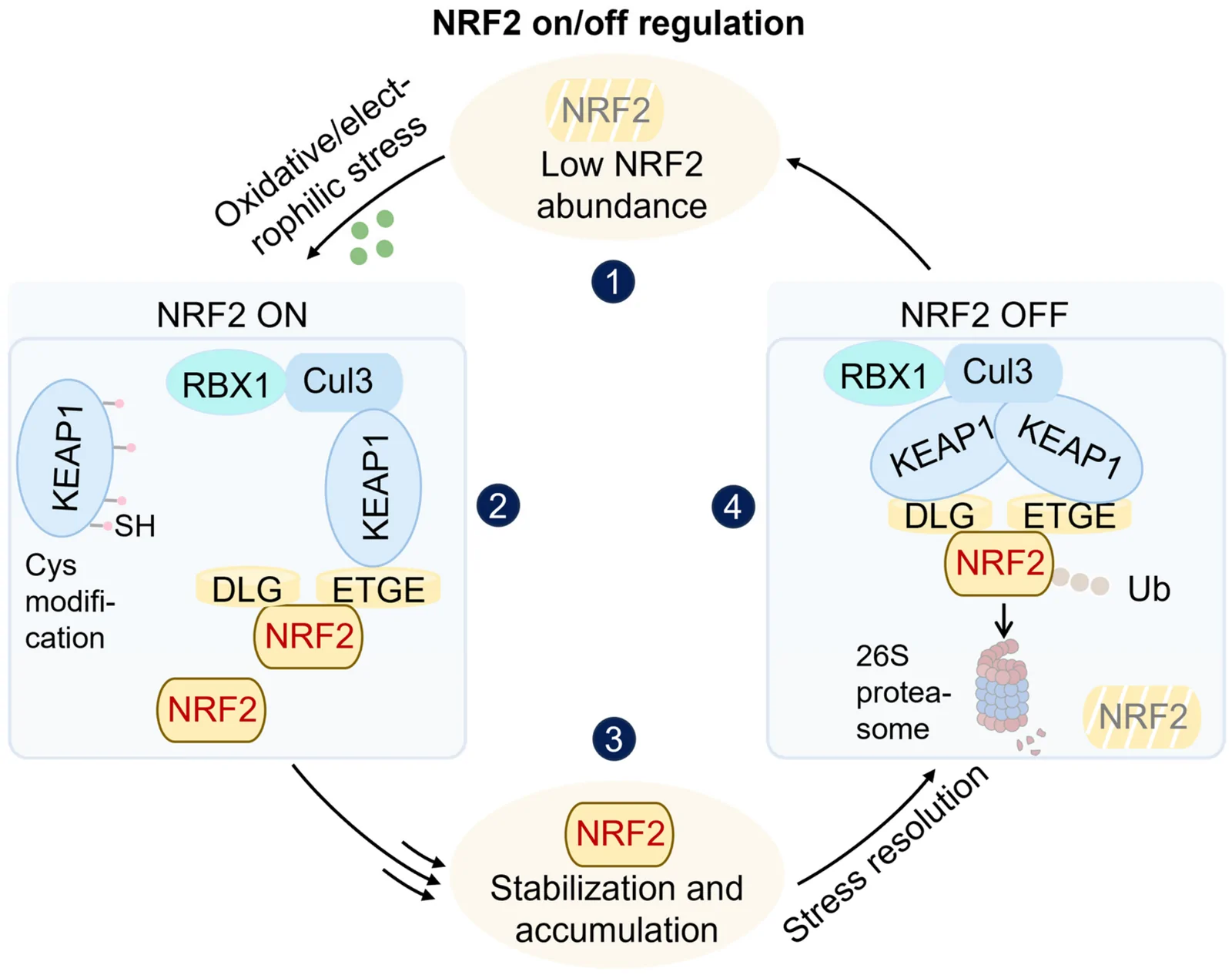
KEAP1-CUL3 controls basal NRF2 degradation and stress-induced stabilization. The KEAP1-CUL3-RBX1 E3 ubiquitin ligase complex maintains low NRF2 abundance under basal conditions through constitutive ubiquitination and proteasomal degradation (NRF2 OFF). Oxidative or electrophilic stress modifies KEAP1 sensor cysteines and transiently impairs NRF2 degradation, allowing newly synthesized NRF2 to stabilize and accumulate (NRF2 ON). After stress resolution, KEAP1-dependent degradation resumes and restores NRF2 to basal abundance.

Together, these reversible mechanisms switch NRF2 signaling on during stress. After stress resolution, KEAP1-dependent degradation resumes, returns NRF2 to low basal abundance, and restores cellular homeostasis (Figure 2).

### 2.2. GSK3/β-TrCP Limits Persistent NRF2 Signaling Through KEAP1-Independent Degradation

GSK3 provides an additional, KEAP1-independent mechanism for controlling NRF2 stability and limiting persistent cytoprotective signaling [10]. Increased GSK3 activity reduced NRF2 abundance and target-gene expression in both KEAP1-deficient mouse embryonic fibroblasts and *KEAP1*-mutant A549 cells, demonstrating that GSK3 can restrain NRF2 independently of KEAP1 [30].

The timing of GSK3-dependent restraint varies with the experimental context. In neuronal oxidative-stress models, early activation of phosphatidylinositol 3-kinase (PI3K)/AKT signaling inhibits GSK3, permitting NRF2 accumulation and activation. As the response progresses, AKT activity declines and GSK3-dependent restraint returns. In these models, delayed negative regulation limits NRF2 persistence after the initial cytoprotective and metabolic response has been established [31,32].

GSK3 regulates NRF2 through two complementary mechanisms. First, the NRF2 Neh6 domain contains two motifs independently recognized by β-TrCP: DSGIS and DSAPGS. GSK3-dependent phosphorylation enhances recognition of DSGIS, whereas recognition of DSAPGS is GSK3-independent. β-TrCP recruits NRF2 to the SCF^β-TrCP^ E3 ubiquitin ligase complex for ubiquitination and proteasomal degradation [30]. Mutation of the GSK3-sensitive serine residues reduces NRF2 ubiquitination and stabilizes NRF2 [33,34]. Second, GSK3 limits NRF2 nuclear residence through FYN. GSK3 promotes FYN nuclear translocation; FYN then phosphorylates NRF2, promoting its nuclear export and subsequent degradation [35].

These mechanisms restrict NRF2 stability and nuclear residence. Alongside constitutive KEAP1-mediated turnover, GSK3-dependent control helps prevent transient adaptation from becoming a persistent pro-survival program.

### 2.3. SMURF2-Mediated Nuclear Clearance Provides Direct Control of NRF2 Signal Termination

Termination of NRF2 signaling also requires nuclear clearance. GSK3-dependent regulation limits NRF2 stability and nuclear residence [6], while SMURF2 directly targets nuclear NRF2 for degradation.

SMURF2 is a HECT (homologous to E6AP C terminus)-type E3 ubiquitin ligase with established roles in regulatory protein turnover [36]. Under cellular stress, SMURF2 translocates to the nucleus and interacts with NRF2 through its HECT domain and the NRF2 Neh1 domain. Its ligase activity promotes K48-linked polyubiquitination and proteasomal degradation of NRF2. Nuclear localization is required because disrupting the SMURF2 nuclear localization signal prevents nuclear NRF2 degradation. Lys555 of human NRF2 was identified as a critical ubiquitin-acceptor site, and the K555R mutation markedly impaired SMURF2-mediated ubiquitination and degradation [7].

SMURF2-mediated nuclear NRF2 degradation limits stress adaptation. Under stress conditions, SMURF2 overexpression reduced the accumulation of ubiquitin/p62-positive protein aggregates and increased cleaved caspase-3 [7], consistent with suppression of an NRF2-dependent proteotoxic response and increased susceptibility to apoptosis. However, these findings, including the NRF2 K555R mutant data, derive from a single recent primary study and have not yet been independently corroborated.

**Figure 3:**
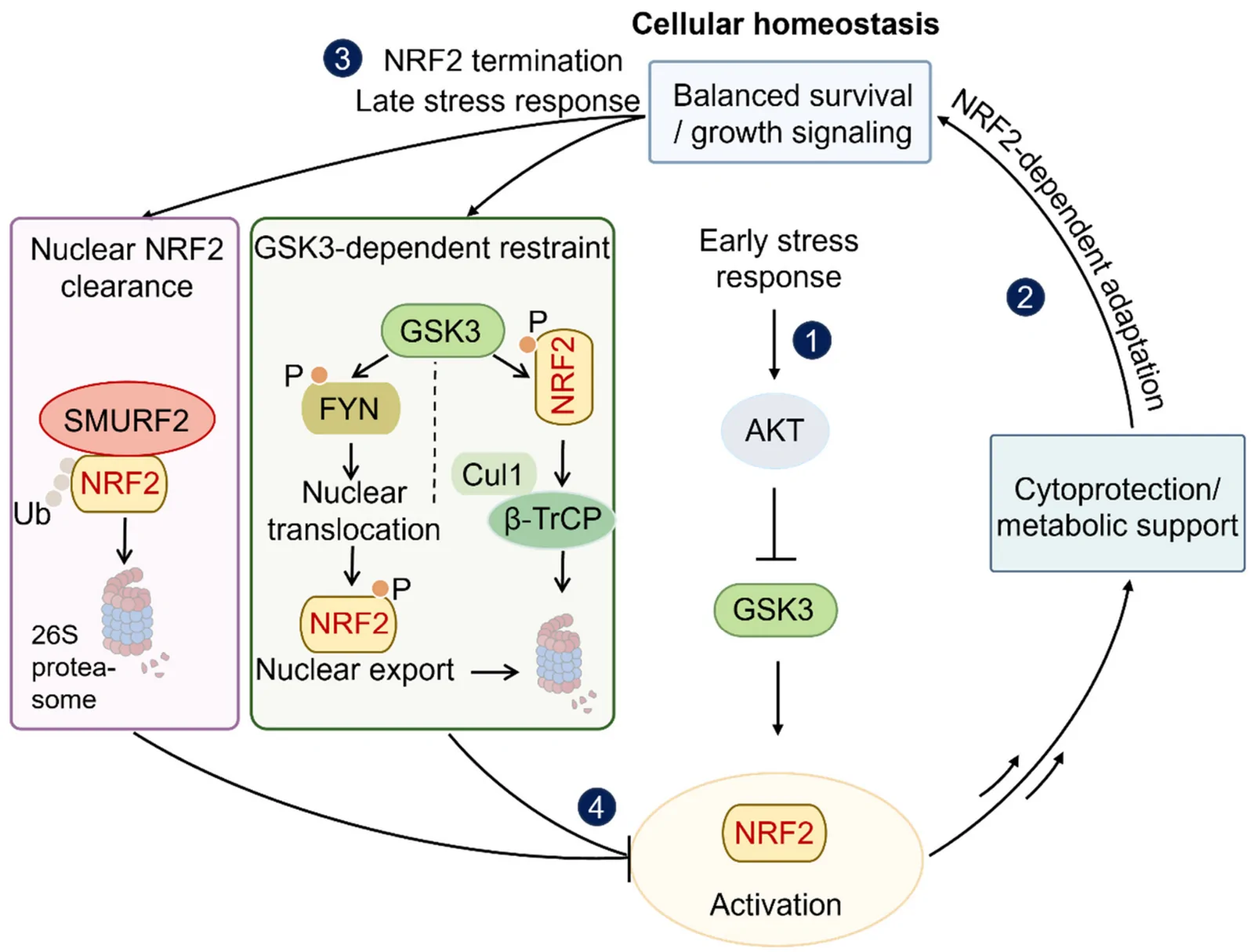
GSK3- and SMURF2-dependent NRF2 regulation contributes to restoration of homeostasis during stress. This context-dependent working model links NRF2 activation to mechanisms that limit its persistence. In neuronal oxidative-stress models, early AKT activation inhibits GSK3, permitting NRF2-dependent cytoprotection and metabolic adaptation; subsequent recovery of GSK3-dependent restraint limits NRF2 persistence. SMURF2 provides an additional stress-inducible mechanism for nuclear NRF2 clearance. The temporal relationship among these regulatory processes requires direct comparative kinetic testing; the numbered steps do not establish a universal temporal sequence.

These degradation mechanisms operate in distinct regulatory and subcellular contexts. KEAP1-CUL3 primarily maintains basal cytoplasmic NRF2 turnover. GSK3-dependent pathways provide additional KEAP1-independent control of NRF2 stability and nuclear residence, whereas SMURF2 directly clears nuclear NRF2. GSK3-dependent regulation and SMURF2-mediated degradation thus converge on limiting persistent NRF2 activity and may contribute to restoring balanced survival and growth signaling (Figure 3). Table 1 compares these pathways with HRD1/SYVN1 and CRL4^DCAF11^.

## 3. NRF2 in Cancer and Increased Stress Tolerance

Cancer cells face persistent oxidative, metabolic, and therapy-induced stress, making sustained adaptation critical for survival [41,42]. Disruption of distinct components of the NRF2 degradation network can result in persistent NRF2 stabilization [43]. Antioxidant defense, metabolic adaptation, and proteostatic programs consequently remain active, increasing stress tolerance and promoting therapeutic resistance [3]. Defective degradation thus converts a normally transient cytoprotective response into a sustained pro-survival state.

### 3.1. KEAP1-CUL3 Disruption Impairs Basal NRF2 Degradation

Loss of constitutive KEAP1-CUL3-dependent degradation is a well-established direct route to sustained NRF2 activity. Somatic loss-of-function mutations in *KEAP1* or *CUL3* disrupt the E3 ligase complex and impair NRF2 ubiquitination and degradation, leading to constitutive activation [44]. Gain-of-function mutations in *NFE2L2* predominantly affect residues in the DLG and ETGE motifs, allowing NRF2 to escape KEAP1 recognition without impairing transcriptional activity [37]. Mutations affecting core KEAP1-NRF2 pathway components, including *KEAP1*, *NFE2L2*, and *CUL3*, have been reported in approximately 15–30% of non-small-cell lung cancers (NSCLCs), with frequencies varying across cohorts and histological subtypes [45,46,47]. *NFE2L2* mutations are enriched in squamous cell carcinoma [48]. In advanced *KRAS*-mutant NSCLC, co-occurring *KEAP1/NFE2L2* alterations were independently associated with shorter overall survival [49].

Nonmutational mechanisms can also weaken basal NRF2 restraint. Epigenetic silencing reduces KEAP1 expression and the capacity for NRF2 degradation [50]. Impaired autophagy promotes p62 accumulation and KEAP1 sequestration, further stabilizing NRF2 [28,29]. Because *SQSTM1* is an NRF2 target gene, this interaction creates a positive feedback loop [51]. The p62-KEAP1-NRF2 axis has been extensively validated in hepatocarcinogenesis and reported in bladder cancer and other malignancies [52,53].

Loss of KEAP1-CUL3-mediated degradation sustains antioxidant and detoxification programs, contributing to tumor growth and resistance to chemotherapy and radiotherapy [37].

### 3.2. GSK3/β-TrCP Disruption Permits Persistent NRF2 Signaling

Oncogenic signaling can prolong NRF2 activity by weakening GSK3-dependent restraint. Loss of phosphatase and tensin homolog (PTEN), a negative regulator of PI3K/AKT signaling, provides a well-defined example. PTEN loss increases PI3K/AKT activity, and AKT-dependent phosphorylation inhibits GSK3. Reduced GSK3 activity decreases phosphorylation of the NRF2 Neh6 degron and subsequent β-TrCP-dependent degradation, thereby stabilizing NRF2 [38,54].

GSK3-dependent degradation therefore links oncogenic signaling to NRF2 stability. Aberrant PI3K/AKT signaling can weaken a KEAP1-independent restraint, prolonging NRF2-dependent cytoprotective programs.

### 3.3. SMURF2 Dysfunction Impairs Nuclear NRF2 Clearance

Loss of SMURF2 impairs nuclear NRF2 clearance and allows cytoprotection to persist. Under oxidative stress, SMURF2 depletion reduced apoptosis. The degradation-resistant NRF2 K555R mutant abolished the increase in cleaved caspase-3 induced by SMURF2-mediated NRF2 degradation, directly linking nuclear clearance to stress-induced apoptosis. Loss of SMURF2 therefore favors an anti-apoptotic state and increases cancer-cell tolerance to oxidative stress [7]. 

SMURF2 loss also promotes proteotoxic adaptation by permitting persistent nuclear NRF2 activity and sustained p62 expression. Increased p62 can sequester misfolded proteins into ubiquitin/p62-positive aggregates, helping cells withstand proteotoxic stress and potentially contributing to chemoresistance. SMURF2-deficient cells also showed increased clonogenic survival after lipopolysaccharide (LPS) exposure [7].

In vivo findings support this mechanism. In xenografts generated from patient-derived glioblastoma cells, SMURF2 knockdown produced larger and heavier tumors after LPS treatment and increased Ki-67 staining. In the NRF2-high/KEAP1-low subgroup of the TCGA-GBMLGG cohort, high SMURF2 expression was associated with improved overall survival than low expression [7]. This association requires caution because survival in the mixed glioma cohort may be influenced by major prognostic factors, including tumor grade and isocitrate dehydrogenase (IDH) mutation status. The experimental findings support SMURF2-mediated nuclear clearance as a restraint on NRF2-driven tumor progression, while the clinical association remains correlative.

Cancer cells can therefore sustain NRF2 activity by weakening distinct controls on its abundance, persistence, and nuclear activity. Genetic or epigenetic disruption of KEAP1-CUL3 removes basal restraint, PTEN loss-driven PI3K/AKT activation weakens GSK3-dependent regulation, and SMURF2 loss compromises nuclear clearance. These defects need not occur together; each can shift NRF2 regulation from transient adaptation toward sustained cytoprotection. Persistent NRF2 activity supports metabolic reprogramming, proteostasis, tumor-cell survival, and resistance to multiple therapeutic modalities.

## 4. Conclusions and Perspectives

The relative contributions of NRF2 regulatory mechanisms are likely to differ across cell types and tissues. Expression of NRF2 and its regulators varies among cell populations. For example, analysis of a published single-nucleus RNA-sequencing dataset from mouse kidney revealed differences in *Nfe2l2*, *Keap1*, and *Gsk3b* expression among renal cell types [55,56]. Variation in SMURF2 abundance may provide another source of regulatory heterogeneity. Higher SMURF2 expression was associated with longer overall survival in the NRF2-high/KEAP1-low subgroup of the reported TCGA-GBMLGG analysis [7]. Whether variation in SMURF2 expression corresponds to differences in nuclear NRF2 clearance among these tumors remains to be established.

The dominant restraint may also change with stress state and duration. GSK3-dependent regulation has been proposed to modulate NRF2 preferentially in stressed or injured tissues rather than under basal conditions [56]. After transient stress resolves, KEAP1 can enter the nucleus and facilitate NRF2 export, allowing KEAP1-CUL3-dependent turnover to resume [57]. During persistent stress, the KEAP1-dependent degradation remains attenuated, and alternative mechanisms may become more important. SMURF2-mediated nuclear NRF2 degradation is stress-inducible [7].

KEAP1, GSK3, and SMURF2 should therefore not be viewed as a fixed hierarchy. Their relative contributions are likely to depend on stress state, regulator abundance, and the persistence of nuclear NRF2. Direct comparative kinetic studies are needed to define their temporal relationships within a single stress response. The evidence base for SMURF2-mediated NRF2 degradation remains more limited than that for KEAP1-CUL3 or GSK3/β-TrCP: the current mechanistic and clinical observations derive from a single recent primary study and await independent corroboration.

These context-dependent differences may have therapeutic implications. Because transient NRF2 activation supports cytoprotection in normal tissues, indiscriminate suppression may be undesirable. Existing NRF2 inhibitors have limitations in specificity and efficacy. Brusatol reduces NRF2 protein levels without directly targeting NRF2; inhibition of protein synthesis may account for this effect. Halofuginone similarly reduces NRF2 through inhibition of proline tRNA synthetase and general protein synthesis. ML385 was initially reported as a direct NRF2 inhibitor targeting the Neh1 domain, but the effectiveness of ML385 as an NRF2 inhibitor remains debated [10,12].

Synthetic-lethal approaches offer an alternative by exploiting dependencies created by constitutive NRF2 activation. NRF2-dependent synthetic lethality has been demonstrated with geldanamycin-derived heat shock protein 90 (HSP90) inhibitors, and UDP-xylose synthase 1 (UXS1) has recently been identified as a selective dependency in *KEAP1*-mutant lung cancer cells [58,59]. These findings provide proof of principle for exploiting NRF2-driven vulnerabilities without directly suppressing NRF2, although selectivity, tumor specificity, and clinical applicability require further validation.

Another strategy is to restore endogenous NRF2 restraint. Inhibition of PI3K/AKT signaling can relieve GSK3 inhibition and promote GSK3/β-TrCP-dependent NRF2 degradation, whereas clobetasol propionate has been shown to promote GSK3/β-TrCP-dependent NRF2 degradation in *KEAP1*-mutant lung cancer models [30,60]. More recently, WS3 was reported to restore GSK3β activity and promote CUL1-β-TrCP-mediated NRF2 degradation, although its selectivity remains unresolved [61].

Therapeutic restoration of SMURF2 remains conceptual. Proposed approaches include enhancing its nuclear activity or mimicking its nuclear NRF2-degrading function while sparing cytoplasmic roles [7]. Because SMURF2 also regulates components of the transforming growth factor-β (TGF-β) pathway, including TGF-β receptors and SMAD proteins, potential effects on these pathways require preclinical evaluation. Restoring endogenous restraint may offer an alternative to indiscriminate NRF2 suppression, but its selectivity and clinical feasibility remain to be established.

Timely termination of NRF2 activity is essential for ensuring that cytoprotective responses remain transient during physiological stress and for restoring cellular homeostasis. KEAP1-CUL3, GSK3/β-TrCP, and SMURF2 collectively restrict NRF2 abundance, persistence, and nuclear activity. In cancer, disruption of this control converts transient adaptation into sustained survival signaling and increases tolerance to oxidative, metabolic, and therapeutic stress. Understanding how NRF2 signaling is restrained and terminated is therefore central to explaining its role in tumor progression.

## Figures and Tables

**Table 1:** Comparison of NRF2 degradation mechanisms.

E3 Ligase System	NRF2 Domain Involved in Recognition	NRF2 Degron or Acceptor Site	Subcellular Compartment	Regulatory Timing	Disruption in Cancer	References
KEAP1-CUL3-RBX1	Neh2	ETGE and DLG	Primarily cytoplasmic	Constitutive degradation under homeostatic	*KEAP1*/*CUL3* loss-of-function; *NFE2L2* gain-of-function mutations	[5,26,37]
SCF^β-TrCP^ (GSK3/β-TrCP axis)	Neh6	DSGIS and DSAPGS; only DSGIS is GSK3-regulated	Subcellular distribution incompletely defined	Basal and stress-responsive; context-dependent	PTEN loss or PI3K/AKT activation reduces GSK3-dependent NRF2 degradation	[30,31,33,38]
SMURF2	Neh1	K555 ubiquitination site	Nuclear	Stress-inducible nuclear degradation	Reduced SMURF2 abundance or activity weakens nuclear NRF2 degradation	[7]
HRD1/SYVN1	Neh4 and Neh5	Not determined	ER-associated	ER stress-associated	TIMP1 overexpression impairs SYVN1-dependent NRF2 degradation in colorectal cancer	[8,39]
CRL4^DCAF11(WDR23)^	Neh2	DIDLID	Cytoplasmic and nuclear isoforms; compartment-specific roles remain unclear	Basal and stress-responsive	Cancer-specific disruption remains incompletely defined	[4,9,40]

The recognition-domain column identifies the NRF2 domain involved, rather than a structural domain of the E3 ligase. Timing is context-dependent and does not imply an obligatory sequence. ER, endoplasmic reticulum.

## Data Availability

No new data were created or analyzed in this study. Data sharing is not applicable to this article.
